# Assessment of Stability of Prothrombin Time, International Normalized Ratio, and Activated Partial Thromboplastin Time Under Different Storage Conditions in Human Plasma

**DOI:** 10.7759/cureus.21268

**Published:** 2022-01-15

**Authors:** Parag Patil, Tushar Sehgal, Priyanka Goswami, Malvika Gaur, Maroof Khan, Shivam Pandey, Sudip K Datta

**Affiliations:** 1 Laboratory Medicine, All India Institute of Medical Sciences, New Delhi, New Delhi, IND; 2 Biostatistics, All India Institute of Medical Sciences, New Delhi, New Delhi, IND; 3 Biostatistics, AIl India Institute of Medical Sciences, New Delhi, New Delhi, IND

**Keywords:** storage temperature, storage time, international normalized ratio, activated partial thromboplastin time (aptt), prothrombin time (pt)

## Abstract

Background

In this study, we aimed to determine the effects of storage time and temperature on commonly performed coagulation tests such as prothrombin time (PT), international normalized ratio (INR), and activated partial thromboplastin time (APTT) in human plasma.

Methodology

Whole blood samples from 100 patients were collected in a 3.2% sodium citrate vacutainer. The blood was centrifuged within two hours of collection at 2,000 g for 10 minutes, and the platelet-poor plasma (PPP) obtained was analyzed for PT, INR, and APTT tests at zero hours (baseline) and repeated at 12 hours, 24 hours, and 36 hours on a fully automated coagulation analyzer at various storage conditions (room temperature, refrigerator, and freezer). The results were categorized into two groups: group 1 comprised results with normal coagulation profile and group 2 comprised results with abnormal coagulation profile. The percentage change of the results from baseline (zero hours) for PT, INR, and APTT tests was also studied. A percentage change of more than ±10% from baseline was considered as a clinically significant change.

Results

In this study, a total of 95 PPP samples were evaluated. The median age of all patients was 44 years (range: 19-65 years). The male-to-female ratio was 0.9:1. The baseline PT, INR, and APTT values were 12.1 seconds, 1.06, and 26.5 seconds, respectively, in group 1, whereas the baseline PT, INR, and APTT values were 19.1 seconds, 1.80, and 36.0 seconds, respectively, in group 2. In the freezer, the samples were stable for PT, INR, and APTT tests at 12 hours, 24 hours, and 36 hours showing a change of <10% from baseline at all three time-points. In the refrigerator, the samples were stable for PT and INR tests for up to 24 hours showing a change of <10% from baseline. In comparison, the samples for the APTT test were not stable at 12 hours, 24 hours, and 36 hours showing a change of 12.1%, 15.5%, and 17.9%, respectively, from the baseline (zero hours). Finally, at room temperature, the samples deteriorated at 12 hours for all coagulation parameters (PT, INR, and APTT).

Conclusions

The patient plasma samples for PT, INR, and APTT tests could be safely stored for up to 36 hours in the freezer. In the refrigerator, samples for PT and INR tests could be safely stored for up to 24 hours while the samples for APTT deteriorated at 12 hours. All patient samples for PT, INR, and APTT tests deteriorated at 12 hours at room temperature.

## Introduction

Prothrombin time (PT) and activated partial thromboplastin time (APTT) are commonly used coagulation tests to assess pathological changes in hemostasis and coagulation systems [1,2]. In addition, the international normalized ratio (INR) is used to monitor oral anticoagulant therapy for reducing the risk of thromboembolic events and minimizing the incidence of bleeding complications [3]. The laboratory testing process is partitioned into three phases, namely, pre-analytical, analytical, and post-analytical phases. The main pre-analytical variables that can affect the results of coagulation tests are the sampling technique, order of draw, type and concentration of anticoagulant, hematocrit, filling status of the sampling tube, transportation, centrifugation, temperature, the time interval between collection and testing, and storage and assay method [1]. The clinical and laboratory standards institute (CLSI) document H21-A5 has recommended that specimens be analyzed within 24 hours for PT and four hours for APTT if stored at room temperature (20-25°C). However, it has not recommended the duration of refrigerated storage (2-8°C). In addition, these guidelines do not mention whether the samples should be kept as whole blood or whether plasma should be separated before storage [1]. Several studies have shown that PT, INR, and APTT determinations may be stable for periods more than the currently recommended guidelines [4,5]. A range of storage times for PT, INR, and APTT from four hours to 24 hours and temperatures ranging from 4°C to 40°C have been studied [1,3]. Furthermore, it has been suggested that each laboratory should establish its acceptable storage times [2].

Another challenge faced by many laboratories during the analytical phase is the maintenance of reliable and accurate results, for which they use quality control (QC) materials provided by the manufacturer. In resource-constrained laboratories, the high cost of these materials is a major concern. A possible way to overcome this problem is to store and reuse patient samples as an internal quality control (IQC) material. CLSI recommends storage at -20°C for two weeks, and storage for extended (greater than two weeks) periods should occur at -70°C or lower [6]. However, the facilities for deep freezer storage (-80°C) are often not available in many laboratories. If sample stability is established in a conventional refrigerator (2-8°C) and freezer (-20° to -16°C), which are generally available in most laboratories, the patient’s plasma samples can be stored easily and reused as IQC material. This may provide an economical solution in helping laboratories with scarce resources to maintain acceptable quality standards. In this study, we aim to determine the stability of patient plasma stored at different times and temperatures in the freezer, in the refrigerator, and at room temperature for PT, INR, and APTT tests to establish our acceptable storage time.

## Materials and methods

This prospective study was performed on the blood samples obtained in the hematology laboratory from both indoor and outdoor patients who attended the central collection facility in the Department of Laboratory Medicine at the All India Institute of Medical Sciences (AIIMS), New Delhi. Ethical clearance was obtained from the Institutional Ethics Committee. The blood samples were collected randomly from 100 patients by a standard venipuncture technique under aseptic precautions in a 2.7 mL vial BD Vacutainer® containing 3.2% buffered sodium citrate solution. All samples that were inadequately filled, hemolyzed, icteric, or lipemic were excluded from the study. All samples were analyzed for PT, INR, and APTT within two hours of sample collection on the Sysmex CS 2000i (Sysmex, Kobe, Japan) automated coagulation analyzer using the photo-optical clot detection method. In this study, to minimize analytical performance variability, we used a single assay kit batch for all analyses. The within and between-batch imprecision was consistent with the manufacturer’s product information. Thromborel® S (International Sensitivity Index: 1.04) reagent was used for the measurement of PT. For the measurement of APTT, calcium chloride (0.025 M) and Dade® Actin® FSL reagent was used. Two levels of controls, namely, normal and abnormal controls, were used as IQC materials. The reference ranges used were as follows: PT: 9.9-13.5 seconds, INR: 0.83-1.25, and APTT: 21.7-29.3 seconds.

The blood samples that were received in the laboratory were centrifuged at 2,000 g for 10 minutes in a tabletop centrifuge to obtain platelet-poor plasma (PPP). PT, INR, and APTT were run on the PPP and the results obtained were taken as baseline values (zero hours). The remaining PPP was aliquoted and stored in nine microcentrifuge tubes (MCT), three each in the freezer, in the refrigerator, and at room temperature to be analyzed at 12 hours, 24 hours, and 36 hours. One MCT from the three MCTs stored in the freezer, in the refrigerator, or at room temperature was analyzed at 12 hours. The same process was repeated at 24 hours and 36 hours. After thawing the samples, the analysis was completed within two hours on the automated coagulation analyzer. All results were categorized into two groups (based on reference interval): group 1 (those with coagulation profile within normal range) and group 2 (those with deranged coagulation profile). The normal coagulation profile was defined as a PT between 9.9 and 13.5 seconds, INR between 0.83 and 1.25, and APTT between 21.7 and 29.3 seconds. The deranged coagulation profile was defined as PT >13.5 seconds, INR >1.25, and APTT >29.3 seconds.

Statistical analysis

All data were summarized with the use of descriptive statistics. Qualitative data were reported with frequencies and percentages. Quantitative data were presented as mean ± SD. Percentage changes in mean values from baseline were also calculated. A change of more than ±10% from baseline was considered as a clinically significant change [1]. Quantitative data were tested for normality using the Kolmogorov-Smirnov test. The Student’s t-test was used to observe the differences between groups if normal, otherwise, the Wilcoxon-rank sum test was applied. To establish the association of categorical data, the chi-square/Fischer exact test was used. All data were summarized and analyzed using STATA (version 16) (StataCorp LP, College Station, TX, USA) software. A p-value of less than 0.05 was considered to represent the statistical significance of the study.

## Results

Of the 100 samples obtained from patients, 95 were included in the analysis. Five samples were excluded from the study due to insufficient volume of PPP. The median age of patients was 44 years (range: 19-65 years). The male-to-female ratio was 0.9:1. For each parameter, a wide range of patient samples both in the normal range (group 1) and abnormal range (group 2) was investigated at various storage conditions (Table 1). Group 1 had 55 patients while group 2 had 40 patients. The baseline PT, INR, and APTT values were 12.1 seconds, 1.06, and 26.5 seconds, respectively, in group 1 whereas the baseline PT, INR, and APTT values were 19.1 seconds, 1.80, and 36.0 seconds, respectively, in group 2.

**Table 1:** Comparison of PT, INR, and APTT in groups 1 and 2 at four time points in different storage conditions. PT: prothrombin time; INR: international normalized ratio; APTT: activated partial thromboplastin time; SD: standard deviation

Parameter	Type of storage	Time of testing in hours	Group 1 [n = 55] Mean ± SD	Group 2 [n = 40] Mean ± SD	P-value
PT (seconds)		0	12.1 ± 0.8	19.1 ± 7.4	<0.0001
	Freezer	12	12.8 ± 1.7	18.3 ± 5.9	<0.0001
		24	13.0 ± 1.7	18.0 ± 5.7	<0.0001
		36	13.5 ± 1.7	19.7 ± 6.4	<0.0001
	Refrigerator	12	12.6 ± 1.9	19.0 ± 6.4	<0.0001
		24	13.3 ± 1.9	19.7 ± 6.6	<0.0001
		36	13.9 ± 2.4	21.0 ± 6.9	<0.0001
	Room temperature	12	14.4 ± 1.8	20.6 ± 6.8	<0.0001
		24	17.1 ± 2.9	24.0 ± 7.2	<0.0001
		36	21.6 ± 7.8	29.9 ± 9.5	<0.0001
INR		0	1.06 ± 0.08	1.80 ± 0.72	<0.0001
	Freezer	12	1.11 ± 2.27	1.69 ± 0.57	<0.0001
		24	1.13 ± 0.14	1.68 ± 0.54	<0.0001
		36	1.21 ± 0.16	1.84 ± 0.62	<0.0001
	Refrigerator	12	1.11 ± 0.18	1.74 ± 0.62	<0.0001
		24	1.17 ± 0.17	1.83 ± 0.64	<0.0001
		36	1.24 ± 0.22	1.94 ± 0.68	<0.0001
	Room temperature	12	1.26 ± 0.16	1.92 ± 0.65	<0.0001
		24	1.52 ± 0.28	2.22 ± 0.69	<0.0001
		36	1.96 ± 0.79	3.30 ± 3.45	0.004
APTT (seconds)		0	26.5 ± 2.0	36.0 ± 8.2	<0.0001
	Freezer	12	27.9 ± 2.9	34.0 ± 8.3	<0.0001
		24	29.6 ± 3.7	33.0 ± 6.9	0.002
		36	29.8 ± 3.4	35.8 ± 8.3	<0.0001
	Refrigerator	12	31.1 ± 3.8	38.5 ± 8.3	<0.0001
		24	32.3 ± 4.0	39.3 ± 7.9	<0.0001
		36	33.1 ± 4.1	39.9 ± 7.9	<0.0001
	Room temperature	12	33.7 ± 4.9	41.3 ± 8.9	<0.0001
		24	37.3 ± 6.2	44.5 ± 9.6	<0.0001
		36	49.2 ± 15.2	52.9 ± 14.7	0.240

Table 2 shows the mean values of coagulation parameters PT, INR, and APTT in the whole cohort in the freezer, in the refrigerator, and at room temperature with the percentage change from baseline (zero hours). First, in the freezer, the samples were stable for all coagulation parameters (PT, INR, and APTT) at 12 hours, 24 hours, and 36 hours showing a change of <10% from baseline at all three time points. Second, in the refrigerator, the samples were stable for both PT and INR tests at 12 hours and 24 hours showing a change of <10% from baseline at both the time points while the samples were not stable at 36 hours showing a difference of 12.3% and 13.0% for PT and INR, respectively. In the refrigerator, the samples for the APTT test were not stable at 12 hours, 24 hours, and 36 hours showing a change of 12.1%, 15.5%, and 17.9%, respectively, from the baseline (zero hours). Finally, at room temperature, the samples deteriorated at all three time points (12 hours, 24 hours, and 36 hours) for all coagulation parameters, viz. PT, INR, and APTT.

**Table 2:** Mean values of PT, INR, and APTT in the whole cohort in the freezer, in the refrigerator, and at room temperature with the percentage change from baseline (zero hours). Note: A change of more than ±10% from baseline was considered clinically significant and is highlighted in bold. PT: prothrombin time; INR: international normalized ratio; APTT: activated partial thromboplastin time; SD: standard deviation

Parameter	Type of storage	Time of testing in hours	Mean ± SD [n = 95] (% change from zero hours)
PT (seconds)		0	15.06 ± 5.9
	Freezer	12	15.11 ± 4.8 (0.3)
		24	15.14 ± 4.5 (0.5)
		36	16.14 ± 5.2 (7.1)
	Refrigerator	12	15.33 ± 5.3 (1.8)
		24	16.06 ± 5.5 (6.6)
		36	16.92 ± 5.9 (12.3)
	Room temperature	12	17.06 ± 5.5 (13.2)
		24	20.03 ± 6.1 (33.0)
		36	25.06 ± 9.4 (66.4)
INR		0	1.30 ± 0.54
	Freezer	12	1.37 ± 1.89 (5.3)
		24	1.31 ± 0.41 (0.7)
		36	1.40 ± 0.48 (7.6)
	Refrigerator	12	1.32 ± 0.48 (1.5)
		24	1.38 ± 0.50 (6.1)
		36	1.47 ± 0.54 (13.0)
	Room temperature	12	1.48 ± 0.50 (13.8)
		24	1.75 ± 0.56 (34.6)
		36	2.40 ± 2.16 (84.6)
APTT (seconds)		0	30.45 ± 7.17
	Freezer	12	30.46 ± 6.45 (0.03)
		24	31.01 ± 5.53 (1.8)
		36	32.28 ± 6.61 (6.0)
	Refrigerator	12	34.14 ± 7.06 (12.1)
		24	35.20 ± 6.81 (15.5)
		36	35.93 ± 6.83 (17.9)
	Room temperature	12	36.88 ± 7.78 (21.1)
		24	40.27 ± 8.55 (32.2)
		36	50.79 ± 15.01 (66.7)

Table 3 shows a comparison of three time points with regards to zero hours (baseline) for coagulation parameters PT, INR, and APTT in group 1 and group 2 at different storage conditions. In group 1, samples stored in the freezer, refrigerator, and room temperature for PT, INR, and APTT tests showed statistically significant differences at 12 hours, 24 hours, and 36 hours at zero hours. In group 2, for the PT test, a statistically significant difference was noted for samples stored in the freezer at 36 hours (p = 0.005) in comparison to zero hours. The difference was also statistically significant for samples stored in the refrigerator and at room temperature at 12 hours, 24 hours, and 36 hours in comparison to zero hours in group 2 for the PT test. For INR, statistically significant differences were noted for samples stored in the freezer at 36 hours (p = 0.004) in comparison to zero hours in group 2. Moreover, the difference was also statistically significant for samples stored in the refrigerator at 24 hours (p = 0.001) and 36 hours (p < 0.0001), as well as for samples stored at room temperature at 12 hours (p < 0.0001), 24 hours (p < 0.0001), and 36 hours (p = 0.004) for INR. For the APTT test, statistically significant differences were noted for samples stored in the refrigerator at 12 hours (p = 0.003), 24 hours (p = 0.001), and 36 hours (p = 0.0004). The difference was statistically significant at room temperature at 12 hours (p < 0.0001), 24 hours (p < 0.0001), and 36 hours (p < 0.0001) in group 2 for the APTT test.

**Table 3:** Comparison of three time points with regards to zero hours (baseline) for coagulation parameters PT, INR, and APTT in groups 1 and 2 at different storage conditions. PT: prothrombin time; INR: international normalized ratio; APTT: activated partial thromboplastin time

Parameters	Type of storage	Time of testing in hours	P-value (Group 1)	P-value (Group 2)
PT (seconds)	Freezer	0 vs. 12	0.004	0.855
		0 vs. 24	0.0002	0.594
		0 vs. 36	0.0001	0.005
	Refrigerator	0 vs. 12	0.0454	0.048
		0 vs. 24	<0.0001	0.001
		0 vs. 36	<0.0001	<0.0001
	Room temperature	0 vs. 12	<0.0001	<0.0001
		0 vs. 24	<0.0001	<0.0001
		0 vs. 36	<0.0001	<0.0001
INR	Freezer	0 vs. 12	0.007	0.817
		0 vs. 24	0.0002	0.675
		0 vs. 36	<0.0001	0.004
	Refrigerator	0 vs. 12	0.045	0.083
		0 vs. 24	<0.0001	0.001
		0 vs. 36	<0.0001	<0.0001
	Room temperature	0 vs. 12	<0.0001	<0.0001
		0 vs. 24	<0.0001	<0.0001
		0 vs. 36	<0.0001	0.004
APTT (seconds)	Freezer	0 vs. 12	0.0001	0.076
		0 vs. 24	<0.0001	0.052
		0 vs. 36	<0.0001	0.645
	Refrigerator	0 vs. 12	<0.0001	0.003
		0 vs. 24	<0.0001	0.001
		0 vs. 36	<0.0001	0.0004
	Room temperature	0 vs. 12	<0.0001	<0.0001
		0 vs. 24	<0.0001	<0.0001
		0 vs. 36	<0.0001	<0.0001

## Discussion

Coagulation tests such as PT, INR, and APTT are widely used and applied in clinical practice; therefore, it is necessary to evaluate the effects of storage temperature and time from the collection on the outcome of these results. Our study investigated the effects of different storage conditions and duration of storage on PT, INR, and APTT tests using patients’ plasma. A summary of a few studies to show the effect of pre-analytical variables, particularly temperature and storage, is shown in Table 4.

**Table 4:** Summary of various studies to show the effect of pre-analytical variables, particularly temperature and storage conditions, on coagulation parameters. PT: prothrombin time; INR: international normalized ratio; APTT: activated partial thromboplastin time; H: heparinized; NH: non-heparinized

Author and reference	Storage Condition	Recommended storage for coagulation tests in hours		
		PT	INR	APTT
Feng et al. [1]	Refrigerator	24	24	12
	Room temperature	24	24	8
Zhao et al. [2]	Refrigerator	24	24	8
	Room temperature	24	24	8
Ikhuenbor et al. [3]	Refrigerator	-	-	-
	Room temperature	2	-	2
Rao et al. [4]	Refrigerator	24	-	12
	Room temperature	24	-	12
Adcock et al. [5]	Refrigerator	24	-	H-4; NH-8
	Room temperature	24	-	H-1; NH-8
Kemkes-Matthes et al. [7]	Refrigerator	-	-	-
	Room temperature	24	-	8
Geelani et al. [8]	Refrigerator	24	-	4
	Room temperature	24	-	4
Oddoze et al. [9]	Refrigerator	-	-	6
	Room temperature	-	-	6
Van Geest-Daalderop et al. [10]	Refrigerator	6	6	-
	Room temperature	6	6	-
Wang et al. [11]	Refrigerator	6	-	6
	Room temperature	4	-	4
Wang et al. [12]	Refrigerator	6	-	-
	Room temperature	8	-	6
Saghir et al. [13]	Refrigerator	4	-	2
	Room temperature	4	-	2
Toulon et al. [14]	Refrigerator	-	-	-
	Room temperature	8	8	8
Present study	Freezer	36	36	36
	Refrigerator	24	24	<12
	Room temperature	<12	<12	<12

Various studies have suggested that acceptable time intervals for coagulation tests can be extended. Feng et al. demonstrated that samples for PT/INR can be safely stored for 24 hours both in the refrigerator and at room temperature, and APTT for 12 hours in the refrigerator and eight hours at room temperature [1]. Zhao et al. found that a storage time interval of up to 24 hours for PT/INR in the refrigerator and at room temperature and 8 hours for APTT either in the refrigerator or at room temperature is acceptable [2]. Kemkes-Matthes et al. reported that PT and APTT can be reliably tested after storage for eight hours at room temperature and that the acceptable time interval can easily be extended to 24 hours for PT [7]. In a study by Geelani et al., it was observed that samples could be safely stored for 24 hours for PT both in the refrigerator and at room temperature, and for APTT for four hours in both the refrigerator and at room temperature [8]. Adcock et al. reported that PT results are stable for up to 24 hours and APTT results are stable for up to eight hours [5]. Rao et al. reported that plasma and whole blood samples can be tested for PT up to 24 hours and APTT for up to 12 hours either at room temperature or in the refrigerator [4]. Moreover, Oddoze et al. have reported that the acceptable time interval for APTT determination is six hours at room temperature or in the refrigerator [9]. Van Geest-Daalderop et al. reported that the acceptable time interval for PT/INR determination is six hours in the refrigerator, at room temperature, and 37°C [10]. In contrast, the studies of Wang et al. and Wang et al. showed that the acceptable time intervals for PT and APTT determination are shorter than those recommended in the guidelines [11,12]. Ikhuenbor et al. concluded that a longer timing (after two hours) from phlebotomy showed a statistically significant increase in the PT and APTT results [3]. In another study by Saghir Sam et al., the PT test was stable with plasma stored at room temperature for four hours, but thereafter, the samples deteriorated, and for APTT, the test results were reliable at two hours and deteriorated thereafter [13]. Toulan et al. showed PT, INR, and APTT were stable with plasma stored at room temperature for eight hours [14]. The differences in the above findings may be explained by factors such as analytical methodologies, automated machines vis-a-vis semi-automated ones, type of reagents, the temperature of surroundings, and other factors.

In this study, we determined the effects of storage in plasma samples on coagulation parameters at different time points and storage conditions (freezer, refrigerator, and room temperature). Not many studies in the literature have studied the effect of freezing (-20°C to -16°C) on plasma samples. We found that in the freezer, the patients’ samples were stable for PT, INR, and APTT tests for 36 hours. In the refrigerator, the samples were stable for PT, INR tests at 12 hours and 24 hours, whereas for APTT, samples were not stable at 12 hours. At room temperature, the samples deteriorated at 12 hours for all coagulation parameters (PT, INR, and APTT). Overall, the samples for the APTT test are less stable than the PT/INR test at both normal and prolonged values. The storage-induced increase of APTT is likely due to the short half-life of coagulation factor VIII [7]. In addition, in this study, we used clinical samples from patients covering a wide range of normal and pathological results to determine the effect of storage time and temperature on PT, INR, and APTT tests in patients’ plasma. The stored samples, especially in the freezer, may be used as an adjunct for the manufacturer’s QC material for PT, INR, and APTT in resource-limited settings. Our findings may be corroborated by further observations based on different study populations, different reagents, and equipment. The limitations of our study were the evaluation of the effects of storage in plasma samples on coagulation parameters at three time points. More time points need to be studied to validate our findings in a larger dataset to establish a gold standard window time.

## Conclusions

Patients’ plasma samples for PT, INR, and APTT tests could be safely stored for up to 36 hours in the freezer. In the refrigerator, samples for PT and INR tests could be safely stored for up to 24 hours while the samples for APTT deteriorated at 12 hours. All patients’ samples for PT, INR, and APTT tests deteriorated at 12 hours at room temperature.
